# Risk factors of fracture following curettage for bone giant cell tumors of the extremities

**DOI:** 10.1186/s12891-022-05447-x

**Published:** 2022-05-19

**Authors:** Shinji Tsukamoto, Andreas F. Mavrogenis, Manabu Akahane, Kanya Honoki, Akira Kido, Yasuhito Tanaka, Davide Maria Donati, Costantino Errani

**Affiliations:** 1 Department of Orthopaedic Surgery, Nara Medical University, 840g, Shijo-cho, Kashihara-city, Nara, 634-8521 Japan; 2 First Department of Orthopaedics, National and Kapodistrian University of Athens, School of Medicine, 41 Ventouri Street, 15562 Holargos, Athens, Greece; 3 Department of Health and Welfare Services, National Institute of Public Health, 2-3-6 Minami, Wako-shi, Saitama, 351-0197 Japan; 4 Department of Rehabilitation Medicine, Nara Medical University, 840, Shijo-cho, Kashihara-city, Nara, 634-8521 Japan; 5 Department of Orthopaedic Oncology, IRCCS Istituto Ortopedico Rizzoli, Via Pupilli 1, 40136 Bologna, Italy

**Keywords:** Giant cell tumor of bone, Curettage, Fracture, Denosumab, Cement, Bone grafting

## Abstract

**Background:**

Following curettage of giant cell tumor of bone (GCTB), it is common to fill the cavity with polymethylmethacrylate (PMMA) bone cement, bone allograft, or artificial bone to maintain bone strength; however, there is a 2–14% risk of postoperative fractures. We conducted this retrospective study to clarify the risk factors for fractures after curettage for GCTB of the extremities.

**Methods:**

This study included 284 patients with GCTBs of the extremities who underwent curettage at our institutions between 1980 and 2018 after excluding patients whose cavities were not filled with anything or who had additional plate fixation. The tumor cavity was filled with PMMA bone cement alone (*n =* 124), PMMA bone cement and bone allograft (*n =* 81), bone allograft alone (*n =* 63), or hydroxyapatite graft alone (*n =* 16).

**Results:**

Fractures after curettage occurred in 10 (3.5%) patients, and the median time from the curettage to fracture was 3.5 months (interquartile range [IQR], 1.8–8.3 months). The median postoperative follow-up period was 86.5 months (IQR, 50.3–118.8 months). On univariate analysis, patients who had GCTB of the proximal or distal femur (1-year fracture-free survival, 92.5%; 95% confidence interval [CI]: 85.8–96.2) presented a higher risk for postoperative fracture than those who had GCTB at another site (100%; *p* = 0.0005). Patients with a pathological fracture at presentation (1-year fracture-free survival, 88.2%; 95% CI: 63.2–97.0) presented a higher risk for postoperative fracture than those without a pathological fracture at presentation (97.8%; 95% CI: 95.1–99.0; *p* = 0.048). Patients who received bone grafting (1-year fracture-free survival, 99.4%; 95% CI: 95.7–99.9) had a lower risk of postoperative fracture than those who did not receive bone grafting (94.4%; 95% CI: 88.7–97.3; *p* = 0.003).

**Conclusions:**

For GCTBs of the femur, especially those with pathological fracture at presentation, bone grafting after curettage is recommended to reduce the risk of postoperative fracture. Additional plate fixation should be considered when curettage and cement filling without bone grafting are performed in patients with GCTB of the femur. This should be specially performed for those patients with a pathological fracture at presentation.

**Supplementary Information:**

The online version contains supplementary material available at 10.1186/s12891-022-05447-x.

## Background

Giant bone tumors of the bone (GCTB) are intermediate-grade primary bone tumors [1]. The standard treatment is usually intralesional curettage, because it can preserve the joint and improve postoperative function. Following curettage of GCTB, it is common to fill the cavity with polymethylmethacrylate (PMMA) bone cement, bone allograft, or artificial bone to maintain bone strength, although there is a 2–14% risk of postoperative fracture [2–4]. Benevenia et al. [2] reported that adding bone grafts near joints when filling with PMMA bone cement could reduce the risk of degenerative osteoarthritis and fractures. The addition of plate fixation is also recommended to prevent fractures after curettage [5, 6]; however, the indications of plate fixation are unclear. Therefore, we conducted this retrospective study to clarify the risk factors for fractures after curettage for GCTB of the extremities.

## Methods

**Fig. 1:**
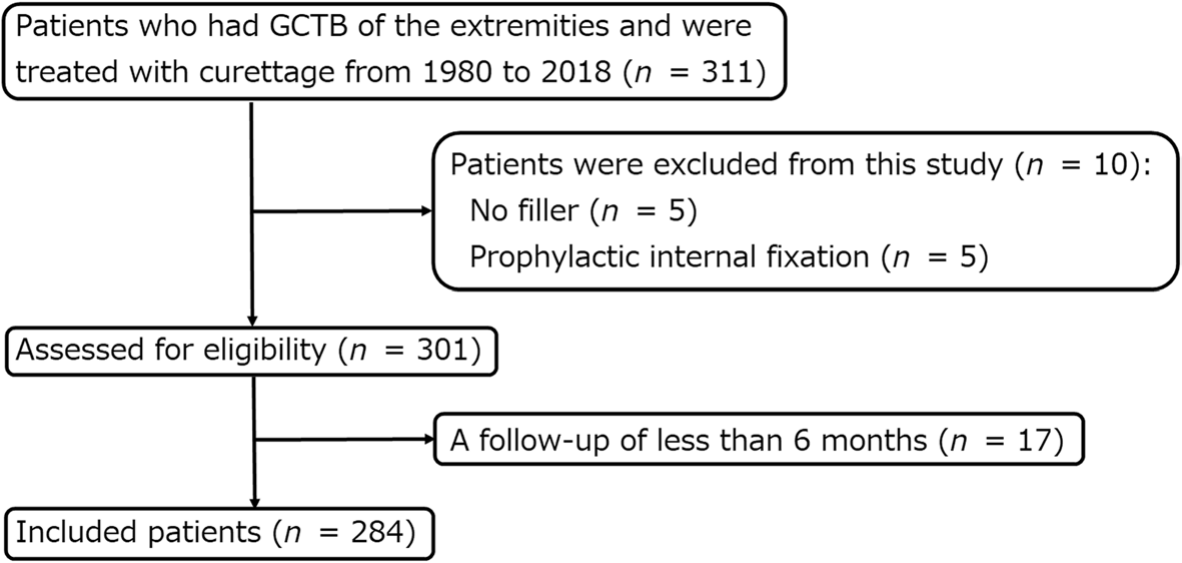
Flow diagram of patients with giant cell tumor of bone of the extremity treated with curettage at three institutions between 1980 and 2018

Of the 311 patients with GCTBs of the extremities who underwent curettage at our institutions between January 1980 and December 2018, 17 with a postoperative follow-up period of less than 6 months, 5 with no filling in the cavity, and 5 with additional plate fixation after curettage were excluded. The remaining 284 patients were included in the study (Fig. 1). Additional plate fixation was performed in patients with large tumors and extensive cortical bone thinning. Additional file 1 shows the details of the five patients who underwent additional plate fixation. The following information was extracted from the medical records: sex, age, tumor site (Because it has been reported that the proximal femur and the distal femur have a high frequency of pathological fractures at presentation, the analysis was performed separately for the femur and other sites [7–10].), Campanacci stage [11], maximum diameter of the tumor located in the distal femur, pathological fracture at presentation, preoperative and postoperative denosumab administration, previous surgery, material filling in the bone defect, local adjuvant therapy, postoperative fracture, local recurrence, and postoperative follow-up period (Table 1).

**Table 1:** Patient characteristics

Variable (***n =*** 284)	No. of patients	Filler		
Sex		PMMA bone cement alone (*n =* 124)	PMMA bone cement and allograft bone grafting (*n =* 81)	Allograft or hydroxyapatite grafting (*n =* 79)
Male	131 (46.1%)	58 (46.4%)	43 (53.1%)	30 (38.5%)
Female	153 (53.9%)	67 (53.6%)	38 (46.9%)	48 (61.5%)
Age (years)				
Median	30	30.7	28.4	29.5
Interquartile range	23.2–40.3	26.0–42.2	21.9–38.3	21.5–40.8
Site				
Distal radius	21 (7.4%)	12 (9.7%)	1 (1.2%)	8 (10.1%)
Proximal femur	13 (4.6%)	1 (0.8%)	5 (6.2%)	7 (8.9%)
Distal femur	94 (33.1%)	56 (45.1%)	28 (34.6%)	10 (12.7%)
Proximal tibia	95 (33.5%)	35 (28.2%)	37 (45.7%)	23 (29.1%)
Distal tibia	16 (5.6%)	5 (4.0%)	4 (4.9%)	7 (8.9%)
Proximal humerus	12 (4.2%)	8 (6.5%)	1 (1.2%)	3 (3.8%)
Others^a^	33 (11.6%)	8 (6.5%)	5 (6.2%)	20 (25.3%)
Campanacci classification				
Stage I	7 (2.5%)	1 (0.8%)	2 (2.5%)	4 (5.1%)
Stage II	220 (77.5%)	101 (80.8%)	60 (74.1%)	59 (75.6%)
Stage III	57 (20.1%)	23 (18.4%)	19 (23.5%)	15 (19.2%)
Pathological fracture at presentation				
No	267 (94.0%)	118 (94.4%)	80 (98.8%)	69 (88.5%)
Yes	17 (6.0%)	7 (5.6%)	1 (1.2%)	9 (11.5%)
Denosumab administration				
No	254 (89.4%)	107 (85.6%)	76 (93.8%)	71 (91.0%)
Yes	30 (10.6%)	18 (14.4%)	5 (6.2%)	7 (9.0%)
Previous surgery				
No	251 (88.4%)	109 (87.2%)	72 (88.9%)	70 (89.7%)
Yes	33 (11.6%)	16 (12.8%)	9 (11.1%)	8 (10.3%)
Local adjuvant therapy				
No	37 (13.0%)	19 (15.2%)	2 (2.5%)	16 (20.5%)
Yes	247 (87.0%)	106 (84.8%)	79 (97.5%)	62 (79.5%)
Fracture				
No	274 (96.5%)	116 (92.8%)	80 (98.8%)	78 (100%)
Yes	10 (3.5%)	9 (7.2%)	1 (1.2%)	0
Local recurrence				
None	217 (76.4%)	102 (81.6%)	65 (80.3%)	51 (65.4%)
≥ 1	67 (23.6%)	23 (18.4%)	16 (19.8%)	27 (34.6%)
Follow-up (months)				
Median	86.5	83.2	94.5	88.3
Interquartile range	50.3–118.8	45.8–101.9	63.6–133.0	45.3–144

*PMMA* Polymethylmethacrylate

^a^Other sites include the distal ulna (7), talus (6), distal humerus (6), calcaneus (3), cuboid (2), hand phalanges (2), foot phalanges (2), proximal ulna (2), proximal radius (1), proximal fibula (1), and metatarsal (1)

Curettage was indicated for GCTB with moderate cortical thinning, well-maintained bone structure, and simple pathological fractures [12–14]. Of the 17 patients who had pathological fractures at presentation, 11 who did not receive preoperative denosumab underwent immobilization using a cast for 4–60 days (median, 7.5 days; IQR, 4.8–18), followed by curettage [15]. The remaining six patients who received preoperative denosumab underwent immobilization using a cast for 120–300 days (median, 150 days), followed by curettage. Curettage was performed through a large cortical bone window with a sharp curette that allowed removal of all visible tumors [12–14]. The cavity was then curetted using a high-speed burr and washed with saline to remove all tumors [12–14]. In 237 patients, phenol was applied to the border of the cavity with cotton-tipped applicators and diluted with alcohol. Cryosurgery using liquid nitrogen spray was performed in three patients, and ablation using an argon beam coagulator was performed in seven patients. The tumor cavity was filled with PMMA bone cement alone (*n =* 124), PMMA bone cement and bone allograft (*n =* 81), bone allograft alone (*n =* 63), or hydroxyapatite graft alone (*n =* 16). The choice to fill the cavity with PMMA bone cement, bone allograft, or hydroxyapatite graft depended on whether or not it was a weight-bearing site (lower limb) and the stability of the remaining cortical bone. PMMA bone cement was used in 31 patients (60.8%) with GCTB of the upper limb, and allograft or hydroxyapatite graft alone was used in 20 patients (39.2%). PMMA bone cement was used in 175 patients (75.1%) with GCTB of the lower limb, and allograft or hydroxyapatite graft alone was used in 58 patients (24.9%). Therefore, PMMA bone cement was used more frequently for GCTB of the lower limb (*p* = 0.038). Bone grafting was performed in 12 patients (92.3%) with GCTB of the proximal femur, whereas it was performed in 38 patients (40.4%) with GCTB of the distal femur (Table 1). Allograft or hydroxyapatite grafting alone was performed significantly more frequently in patients with a pathological fracture at presentation (*p* = 0.015) (Table 1). The maximum diameter of the tumor was measurable in 42 of the 94 patients with GCTB of the distal femur. The tumor size in the PMMA bone cement alone group was significantly smaller than that in the bone grafting group (median 6 cm [IQR, 5–6.8] vs. median 7 cm [IQR, 7–8], *p* = 0.001). The subchondral region is frequently protected with allograft bone chips when the cavity has a thin subchondral bone following curettage.

**Fig. 2:**
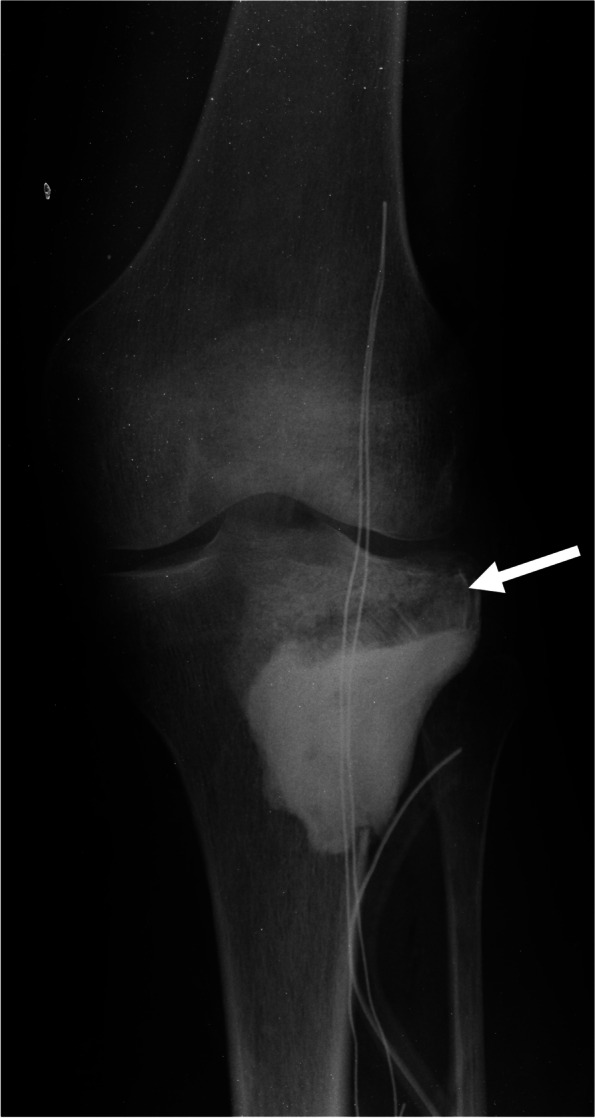
Radiography after PMMA bone cement filling following allograft chip bone filling in the subchondral region after curettage. The arrow indicates the allograft chip bone

**Fig. 3:**
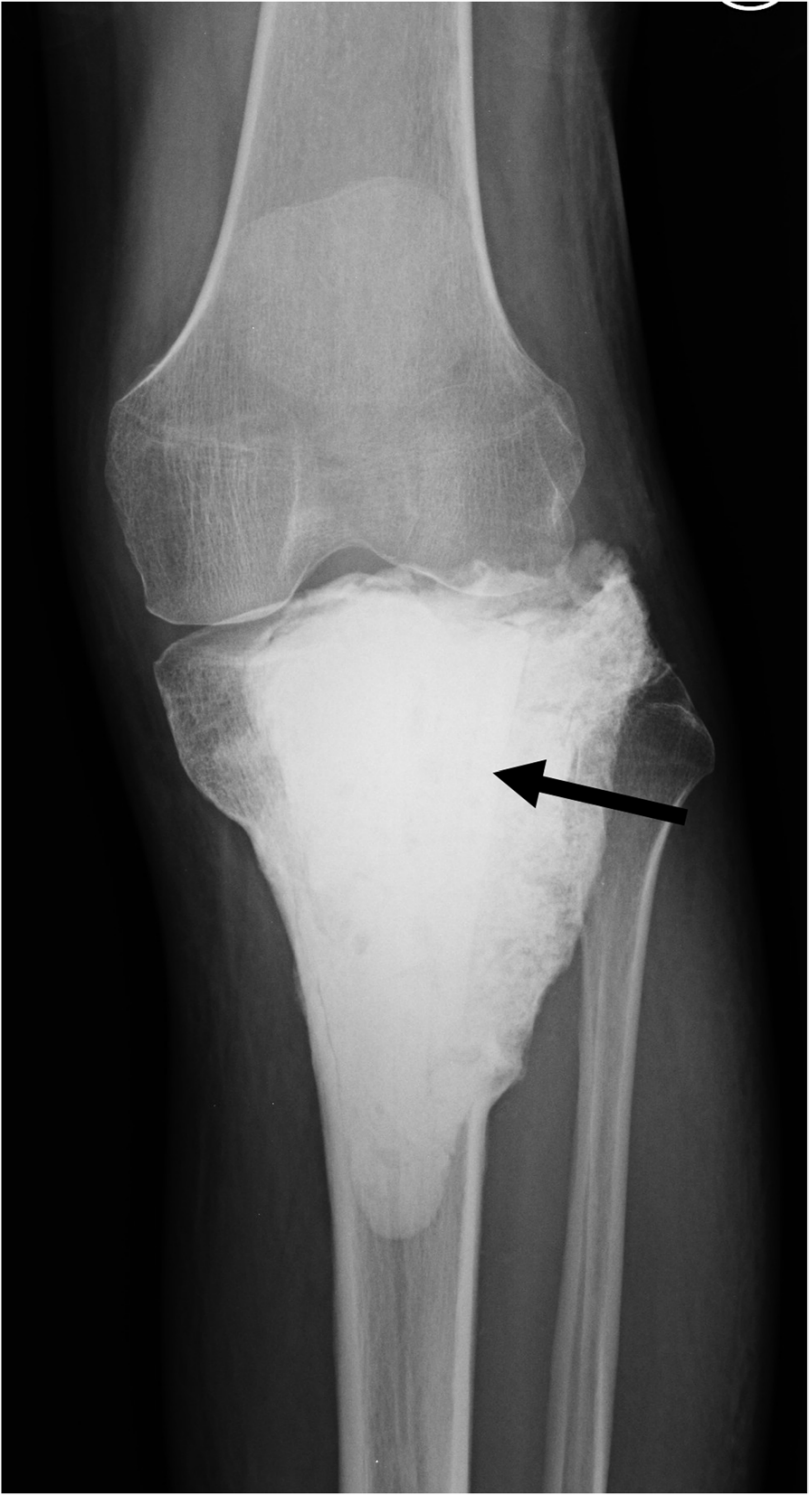
Radiography after cement filling following large cortical bone allograft insertion after curettage. The arrow indicates the large cortical bone allograft

**Fig. 4:**
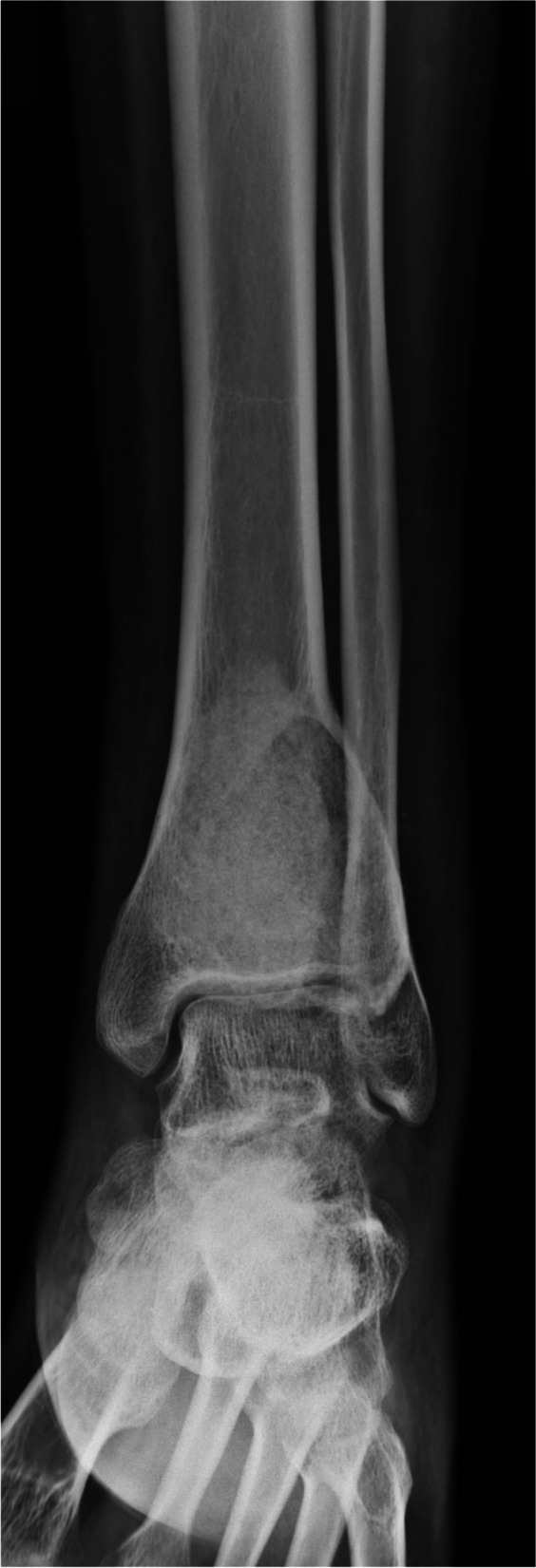
Radiography after filling allograft chip bone following curettage

Reconstruction using PMMA bone cement with a bone allograft was performed in two different ways. First, PMMA bone cement was filled after filling the allograft bone chips into the subchondral lesion to protect articular cartilage from the thermal effect of PMMA bone cement (71 patients)(Fig. 2). Second, the PMMA bone cement was filled after inserting a large cortical bone allograft to support the articular surface (10 patients) (Fig. 3). For bone allograft reconstruction, allograft bone chips, with or without cortical bone allografts, were inserted into the cavity (Fig. 4).

Denosumab was used particularly in patients with GCTB of the distal radius (for tumor downstaging) because the risk of local recurrence in this location is higher than that in other sites [16]. Preoperatively, 120 mg of denosumab was administered subcutaneously once a week for 1 month and then once a month for 1–7 months. Postoperatively, the same dose was administered once a month for 1–7 months.

Postoperatively, the patients who underwent curettage with PMMA bone cement were allowed to bear weight in the first week. Patients who had a pathological fracture at presentation that was later filled with PMMA bone cement were kept non-weight-bearing for approximately 4 weeks, followed by partial weight-bearing and then full weight-bearing 12 weeks after surgery. In contrast, patients treated with curettage without PMMA bone cement were not allowed until after 4 weeks, and were then allowed to bear partial weight until their radiographs showed sufficient consolidation to allow full weight-bearing, which was usually achieved in 12 weeks.

Routine follow-up examinations were performed every 4 months for the first 2 years, every 6 months for the next 3 years, and annually thereafter. Follow-up evaluations included radiography of the tumor area and computed tomography of the chest. Local recurrence and surgery-related complications were recorded.

The chi-square test was used to evaluate the association between the two variables. The difference between the two independent samples was statistically analyzed using the Mann–Whitney U test for nonparametric analyses. Fracture-free survival was defined as the interval between the first curettage and postoperative fracture or the last follow-up. Fracture-free survival was evaluated using Kaplan–Meier survival analysis, and survival curves were compared using a log-rank test. Statistical significance was set at *P* < 0.05. Analyses were performed using JMP 14 software (SAS Institute Inc., Cary, NC, USA).

Informed consent was obtained from each participant in IRCCS Istituto Ortopedico Rizzoli and National and Kapodistrian University of Athens, School of Medicine. Nara Medical University Ethics Committee approved a waiver for the informed consent. The study was conducted in accordance with the guidelines of the Declaration of Helsinki and approved by IRCCS Istituto Ortopedico Rizzoli di Bologna Ethics Committee and Nara Medical University Ethics Committee. Ethics committee approval was not necessary for retrospective studies at National and Kapodistrian University of Athens, School of Medicine.

## Results

Fractures after curettage occurred in 10 (3.5%) patients, and the median time from the curettage to fracture was 3.5 months (interquartile range [IQR], 1.8–8.3 months). The median postoperative follow-up period was 86.5 months (IQR, 50.3–118.8 months). Local recurrence occurred in 67 (23.6%) patients, and the median time from the first curettage to local recurrence was 15.4 months (IQR, 10.8–29.6 months).

**Fig. 5:**
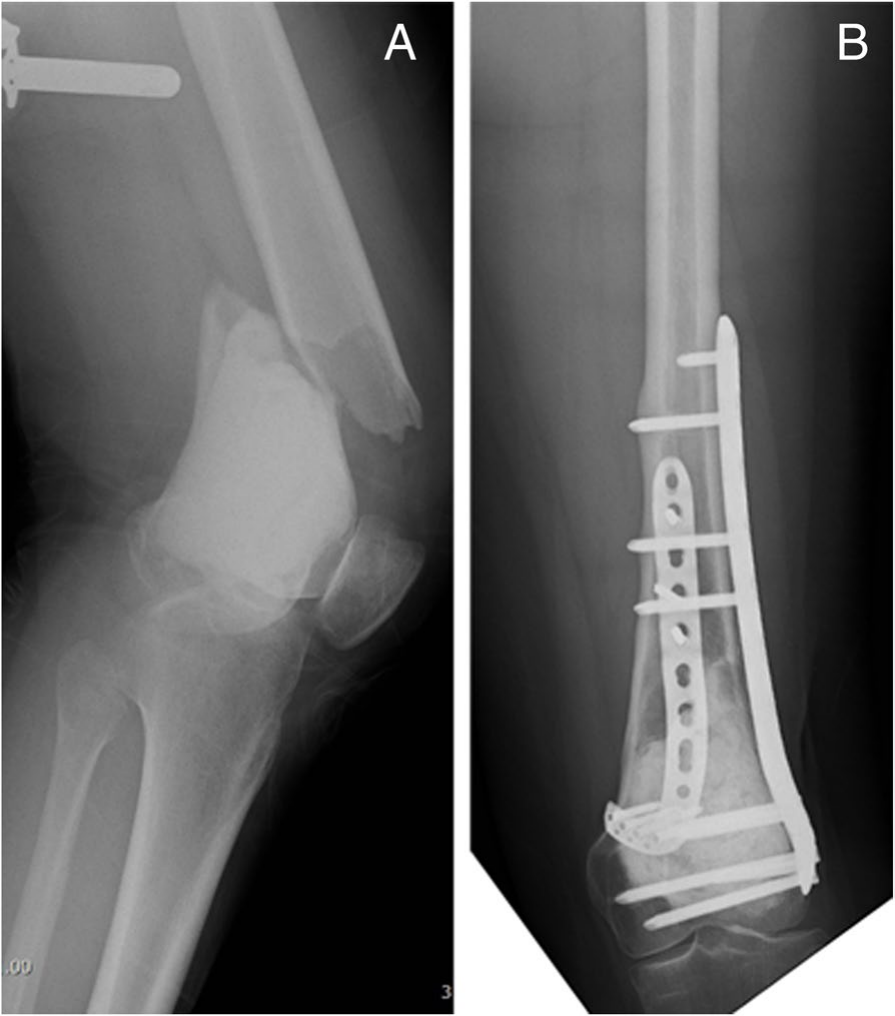
**A,** Radiography at the time of fracture (Case 10, Table 2). **B,** Radiography 3 years and 8 months after open reduction and internal fixation showed bone union

**Fig. 6:**
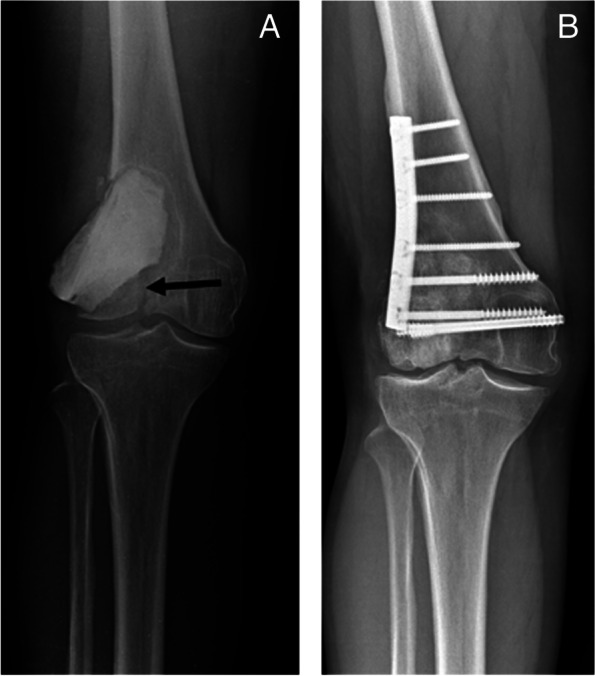
**A,** Radiography at the time of fracture. The arrow indicates the fracture line (Case 6, Table 2). **B,** Radiography 2 years after cement removal, filling of allografts bone chips, and osteosynthesis with a plate

Table 2 shows the details of the ten patients with postoperative fractures. Six patients were able to preserve their joints after undergoing osteosynthesis, and all showed bone union (Fig. 5). Meanwhile, four patients underwent *en bloc* resection, including the joints, and needed reconstruction with a prosthesis or massive bone allograft (Fig. 6). Nine of the ten patients with postoperative fractures developed GCTB in the distal femur (Table 2). The maximum diameter of the tumor was measurable in 42 of the 94 patients with GCTB of the distal femur. Of the 42 patients, four with postoperative fractures had a median tumor size of 6 cm (IQR, 6.0–7.9 cm), whereas 38 without postoperative fractures had a median tumor size of 6.8 cm (IQR, 5.5–7.5). There was no significant difference between the two groups (*p* = 1.0).

**Table 2:** Details of 10 patients with postoperative fracture

Case	Sex	Age	Site	Campanacci classification	Pathological fracture at presentation	Denosumab administration	Previous surgery	Surgery	Local adjuvant therapy	Time to fracture (months)	Treatment for postoperative fracture	Local recurrence	Follow-up period (months)
1	M	29	Distal femur	Stage II	No	No	No	PMMA bone cement alone	Phenol	4	Resection and reconstruction with a hemicortical bone allograft	No	76
2	F	21	Distal femur	Stage II	No	No	No	PMMA bone cement alone	Phenol	15	Cement removal, filling of allografts bone chips, and osteosynthesis with a plate	No	80
3	M	61	Distal femur	Stage II	Yes	No	No	PMMA bone cement alone	Phenol	1	Osteosynthesis with a plate	No	57
4	F	32	Proximal tibia	Stage II	No	No	Yes	PMMA bone cement alone	Phenol	42	A screw fixation for a detachment fracture of tibial tuberosity	No	99
5	F	29	Distal femur	Stage II	No	No	No	PMMA bone cement alone	Phenol	6	Resection and reconstruction with a prosthesis	No	74
6	F	29	Distal femur	Stage II	No	No	No	PMMA bone cement alone	Phenol	2	Cement removal, filling of allografts bone chips, and osteosynthesis with a plate	No	25
7	M	29	Distal femur	Stage II	No	No	No	PMMA bone cement alone	None	6	Resection and reconstruction with a hemicortical bone allograft	No	93
8	M	35	Distal femur	Stage III	No	No	No	PMMA bone cement and allograft bone grafting	Phenol	3	Resection and reconstruction with a prosthesis	No	51
9	F	67	Distal femur	Stage II	Yes	Yes	No	PMMA bone cement alone	None	3	Radiofrequency ablation and percutaneous cementation for local recurrence and fracture	Yes	40
10	F	30	Distal femur	Stage II	No	No	No	PMMA bone cement alone	Cryosurgery	1	Osteosynthesis with a plate	No	44

*M* Male, *F* Female, *PMMA* Polymethylmethacrylate

**Fig. 7:**
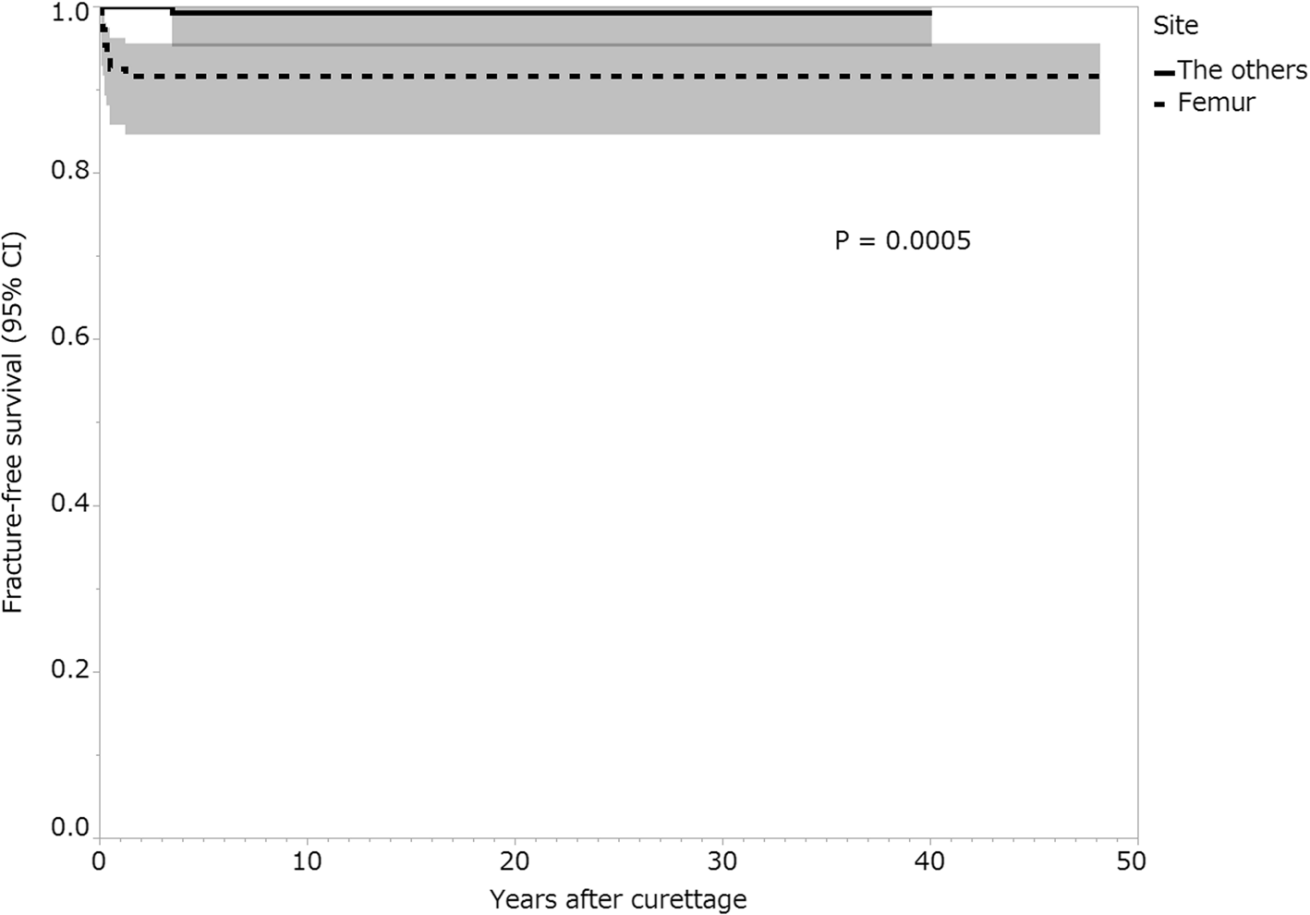
Fracture-free survival rates of patients by tumor site. Shading around the curves represents the 95% confidence intervals (CI)

**Fig. 8:**
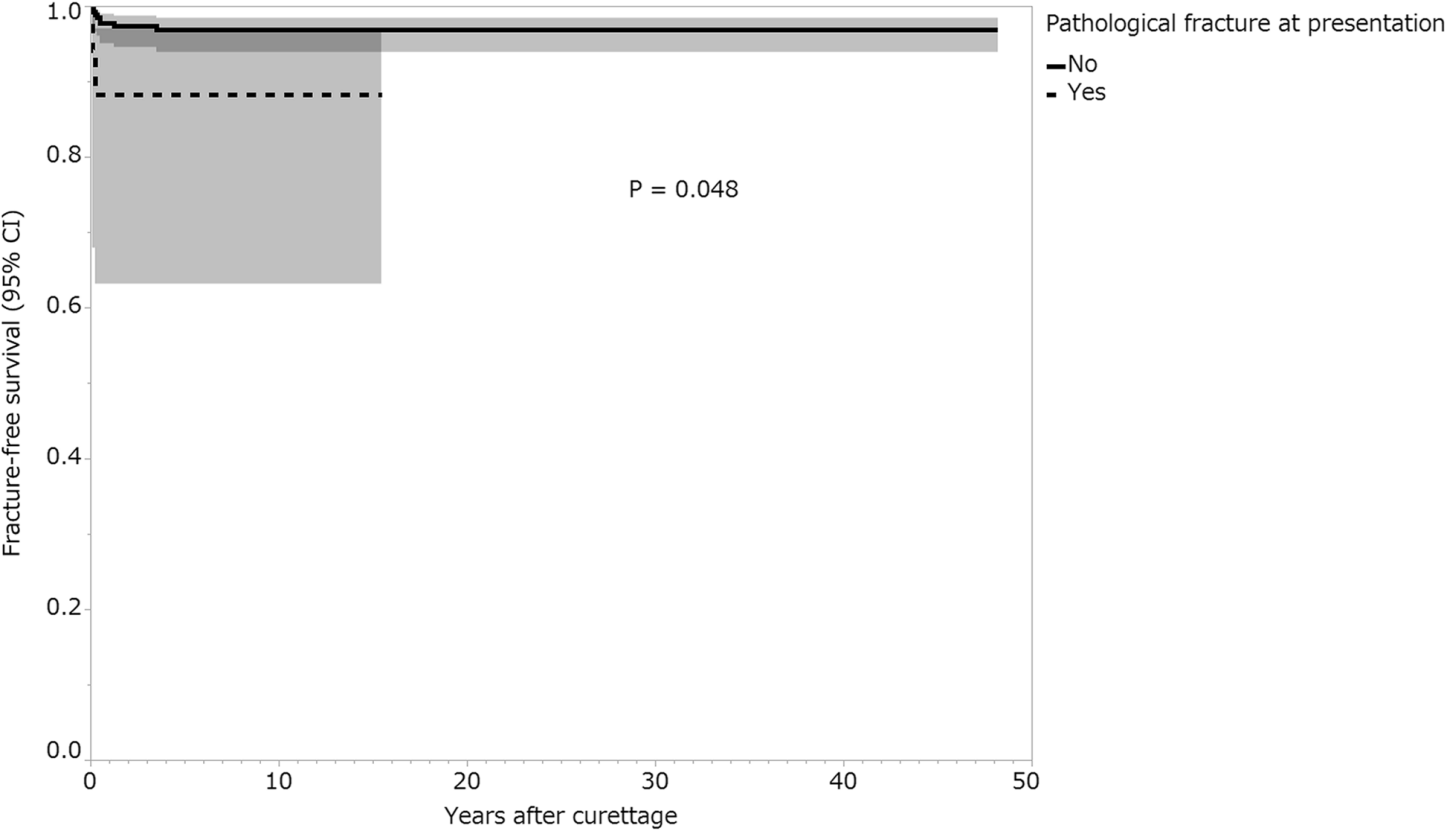
Fracture-free survival rates of patients by pathological fracture at presentation. Shading around the curves represents the 95% confidence intervals (CI)

**Fig. 9:**
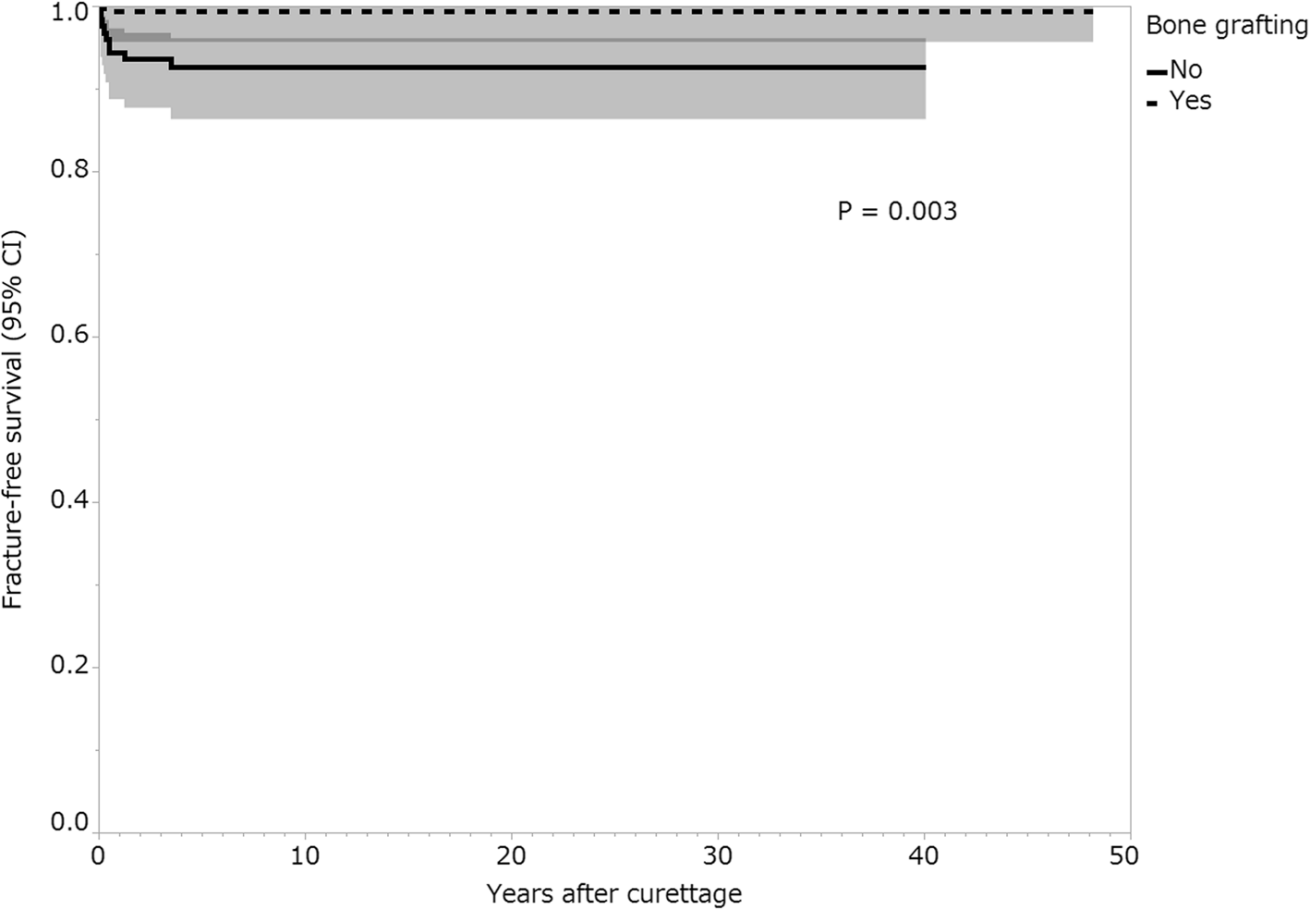
Fracture-free survival rates of patients by use of bone grafting. Shading around the curves represents the 95% confidence intervals (CI)

On univariate analysis, patients with GCTB of the proximal or distal femur (1-year fracture-free survival, 92.5%; 95% confidence interval [CI]: 85.8–96.2) presented a higher risk for postoperative fracture than patients who had GCTB at another site (100%; *p* = 0.0005; Fig. 7; Table 3). Fracture rates after curettage were 0% (0/13 patients) and 9.6% (9/94 patients) in the GCTB of the proximal femur and distal femur, respectively. Patients with a pathological fracture at presentation (1-year fracture-free survival, 88.2%; 95% CI: 63.2–97.0) presented a higher risk of postoperative fracture than patients without a pathological fracture at presentation (97.8%; 95% CI: 95.1–99.0; *p* = 0.048; Fig. 8; Table 3). Patients who received bone grafting (1-year fracture-free survival, 99.4%; 95% CI: 95.7–99.9) had a lower risk of postoperative fracture than those who did not receive bone grafting (94.4%; 95% CI: 88.7–97.3; *p* = 0.003; Fig. 9; Table 3). Univariate analysis revealed no association between the following variables and postoperative fractures: sex, age, Campanacci stage, preoperative and postoperative denosumab administration, previous surgery, and local adjuvant therapy (Table 3).

**Table 3:** Univariate analysis for fracture-free survival in patients who received curettage for GCTBs of the extremities

Variable	No. of patients (***n =*** 284)	1-year fracture-free survival (95% CI) (%)	***P*** value
Sex			0.707
Male	131	96.9 (92.1–98.8)	
Female	153	97.4 (93.2–99.0)	
Age (years)			0.978
< 30	140	97.1 (92.6–98.9)	
≥ 30	144	97.2 (92.8–99.0)	
Site			0.0005^a^
Femur	107	92.5 (85.8–96.2)	
Others	177	100.0	
Campanacci classification			0.424
Stage I, II	227	96.9 (93.7–98.5)	
Stage III	57	98.2 (88.6–99.8)	
Pathological fracture at presentation			0.048^a^
No	267	97.8 (95.1–99.0)	
Yes	17	88.2 (63.2–97.0)	
Denosumab administration			0.952
No	254	97.2 (94.3–98.7)	
Yes	30	96.7 (79.8–99.5)	
Previous surgery			0.858
No	251	96.8 (93.8–98.4)	
Yes	33	100.0	
Bone grafting			0.003^a^
No	125	94.4 (88.7–97.3)	
Yes	159	99.4 (95.7–99.9)	
Local adjuvant therapy			0.502
No	37	94.6 (80.8–98.6)	
Yes	247	97.6 (94.7–98.9)	

^a^Statistically significant. *GCTB* Giant cell tumor of bone, *CI* Confidence interval

## Discussion

Fractures after curettage of GCTBs of the extremities are serious complications that usually require surgery. This study revealed that the risk of fracture after curettage increased in patients with GCTB of the femur, especially in those with pathological fracture at presentation, and that the use of bone allografts after curettage reduced the risk of postoperative fracture.

In our study, GCTBs of the femur were associated with an increased risk of fracture after curettage. No previous studies have investigated the correlation between tumor site and risk of postoperative fracture after curettage, but it has been reported that the frequency of pathological fractures at presentation is high in GCTB of the femur [7–10]. Our results confirm these findings.

GCTB with pathological fractures at presentation had an increased risk of fracture after curettage. Van der Heijden et al. retrospectively analyzed 48 patients with GCTB (27 of whom had GCTB of the femur) with pathological fractures at presentation and reported that the major complication rate was 4% after curettage with adjuvants (one of 23) and 16% after *en bloc* resection (4 of 25 patients) [17]. One major complication of the curettage group was a nonunion. The patient, who had GCTB of the distal femur with a pathological fracture at presentation, underwent immediate curettage and cement-filling but developed nonunion, requiring iliac bone graft and cast fixation [17]. The scoring system for surgical guidelines for GCTB around the knee by the Chinese expert consensus includes pathological fracture, cortical bone destruction, tumor size, and articular surface involvement [18]. The total scores ranged from 1 to 12 points: a total score of 1–4 suggested curettage alone, 5–9 points indicated intralesional curettage with internal fixation for fewer surgery-related complications, and 10–12 points indicated prosthesis replacement for long-term local control [18].

The risk of fracture after curettage was lower in the bone grafting group than that in the PMMA bone cement alone group. Benevenia et al. [2] also reported that of 21 patients in the cement with bone graft group, one experienced a periarticular fracture (5%), while of 22 patients in the cement without bone graft group, five experienced periarticular fractures (23%). In contrast, Wallace et al. performed curettage with cement or bone grafting in 36 skeletally immature patients with locally aggressive bone tumors [19]. There were no postoperative fractures in the 17 patients reconstructed with cement, while there were 3 fractures in the 19 patients reconstructed with bone graft, without a statistically significant difference [19]. Furthermore, PMMA bone cement is harder than subchondral bone and cartilage; thus, it concentrates pressure on thin cartilage and subchondral bone [20, 21]. PMMA is non-biodegradable and cannot be biologically integrated into the surrounding host bone [22]. Welch et al. described a sclerotic rim created by increased new trabecular bone formation, separating the cement from the surrounding bone and subchondral bone layers [22]. This sclerotic rim can decrease the shock-absorbing capacity of the subchondral bone layer [22]. In addition, thermal necrosis of the subchondral bone and articular cartilage can occur when PMMA bone cement fills a subchondral defect [20, 23]. PMMA bone cement can cause cartilage damage, fractures, and degenerative osteoarthritis [3, 20, 23].

Bone grafts are commonly used as fillers [24, 25]. Animal model experiments have demonstrated that the subchondral strength of defects filled with cancellous bone is slightly greater than that of empty defects [20, 25]. Consequently, there is an increased likelihood of subchondral bone collapse and fracture after bone grafting. However, after complete bone remodeling, the strength of the subchondral bone is fully restored. Animal model experiments showed reduced subchondral strength in both the bone graft and PMMA bone cement groups at 3 weeks; however, after 12 weeks, the bone graft group returned to normal, and the PMMA bone cement group recovered only up to 79% of the normal contralateral limb [24].

The results of this study showed no correlation between fracture risk after curettage and tumor size in GCTB of the distal femur. Hirn et al. [26] investigated the postoperative fracture risk (14 patients) in 146 patients who underwent curettage alone and with no filler for benign bone tumors in the distal femur or proximal tibia. The risk of fracture was 5% in patients with a bone defect volume of less than 60 cm^3^ compared with 17% in patients with a bone defect volume greater than 60 cm^3^ (*p* = 0.01). In addition, the risk of fracture was 3% when the maximum diameter of the bone defect was 5 cm or less, compared with 15% when the diameter was greater than 5 cm (*p* = 0.02). Jeys et al. performed a retrospective study of 54 patients with GCTB of the distal femur and reported that a ratio of tumor volume to distal femoral volume of 54% or higher was associated with an increased risk of pathological fracture [27]. Amanatullah et al. created a model of a bone defect in the distal femur of a cadaver and conducted a biomechanical study to examine the relationship between the defect size and fracture under torsional stress. They reported an increased risk of fractures with defect sizes that destroyed more than 50% of the cortical width [28]. Human cadaveric distal femoral finite element analyses showed that when the bone defect was 35% or more of the epiphyseal volume, the bone strength decreased, and was greater in the medial defect than in the lateral defect [29, 30]. Furthermore, the proximal end of the cortical window and the interior wall of the bone-cement interface were the most vulnerable sites [29, 30].

Denosumab is a monoclonal antibody that binds to the receptor activation of nuclear factor-kappa β ligand, which stops bone destruction and hardens lesions [31]. It has been reported that tumor cells are hidden in osteosclerotic lesions and that leaving them behind leads to recurrence [32, 33]; however, the effect of osteosclerosis on preventing fractures after curettage was not observed in this study. In this study, no correlation was observed between local adjuvant therapy and fracture risk. Compared to liquid nitrogen, phenol has less penetration into the bone (10–20 mm vs. 0.2 mm), and therefore, has a lower fracture rate [34, 35]. Bombardier et al. provided various local adjuvant therapies and subsequently performed histological evaluations of bone defect models of porcine humeri and femora [36]. They reported that the average depth of necrosis was 0.3 mm in the phenol group and 2.54 mm in the liquid nitrogen spray group [36]. Van der Heijden et al. reported that the use of liquid nitrogen significantly increased the risk of non-oncological complications, including osteoarthritis, infection, postoperative fracture or femoral condyle collapse, nonunion, nerve palsy, and PMMA leakage [4]. Because phenol was used in most cases, there may not have been a correlation between local adjuvant therapy and fracture risk in this study.

Regarding the treatment of fractures after curettage, all 6 patients who underwent osteosynthesis were able to obtain bone union and preserve the joint in this study. Pritsch et al. [37] did not recommend osteosynthesis because eight patients who had fractures after curettage and cryosurgery using the direct pour technique underwent osteosynthesis, but seven did not achieve bone union [37]. Therefore, cryosurgery using the direct-pour technique may significantly reduce bone union. Cryosurgery using liquid nitrogen spray has been reported to reduce fracture rates compared to the direct pour technique (0% vs. 17%) [38]; therefore, cryosurgery using liquid nitrogen spray may preserve bone union ability. In contrast, Hirn et al. reported that only 2 of 14 patients with fractures after curettage without local adjuvant therapy required internal fixation, and the rest could be treated with conservative therapy [26].

Our study had several limitations. This was a retrospective study and had an indication of bias for each treatment. Because the number of patients who had fractures after curettage was as small as 10, multivariate analysis was not possible, and confounding factors could not be corrected. Type 2 error due to the small sample size is possible. If an adequate number of patients is enrolled in the future, significant differences may appear regarding the other variables in this study. In future studies, it will be necessary to increase the number of cases and perform multivariate analyses.

## Conclusions

For GCTBs of the femur, especially those with pathological fractures at presentation, bone grafting after curettage is recommended to reduce the risk of postoperative fractures. Additional plate fixation should be considered when curettage and cement filling without bone grafting are performed in patients with GCTB of the femur, especially for those patients with pathological fractures at presentation.

## Supplementary Information

**Additional file 1.**

## Data Availability

The datasets generated, analyzed, or both during the present study are not publicly available to protect the privacy of participants but are available from the corresponding author upon reasonable request.

## Abbreviations

GCTB: Giant bone tumor of bone
PMMA: Polymethylmethacrylate
IQR: Interquartile range
CI: Confidence interval
