# Supplementary material for: Risk factors of fracture following curettage for bone giant cell tumors of the extremities
Source: BMC Musculoskelet Disord. 2022 May 19;23:477. doi: 10.1186/s12891-022-05447-x (PMC9118605; doi:10.1186/s12891-022-05447-x)
Supplement: Supplementary file 1 — Additional file 1. [file 12891_2022_5447_MOESM1_ESM.docx]

| **Appendix 1. Details of five patients with additional plate fixation** | | | | | | | | | | | | | |
| --- | --- | --- | --- | --- | --- | --- | --- | --- | --- | --- | --- | --- | --- |
| **Case** | **Sex** | **Age (years)** | **Site** | **Campanacci classification** | **Tumor size (cm)** | **Pathological fracture at presentation** | **Denosumab administration** | **Previous surgery** | **Surgery** | **Local adjuvant therapy** | **Postoperative fracture** | **Local recurrence** | **Follow-up period (months)** |
| 11 | F | 30 | Distal femur | Stage III | 7.5 | No | No | No | PMMA bone cement, allograft bone grafting, and plate fixation | Phenol | No | No | 82 |
| 12 | F | 24 | Distal femur | Stage II | 7 | No | No | No | PMMA bone cement and plate fixation | Phenol | No | No | 82 |
| 13 | M | 43 | Distal femur | Stage II | 10 | No | Yes | No | PMMA bone cement, allograft bone grafting, and plate fixation | None | No | Yes | 98 |
| 14 | F | 25 | Distal femur | Stage III | 8.5 | No | No | No | PMMA bone cement and plate fixation | Phenol | No | Yes | 93 |
| 15 | F | 33 | Distal femur | Stage II | Unknown | No | No | No | PMMA bone cement and plate fixation | Phenol | No | No | 91 |

M, male; F, female; PMMA, polymethylmethacrylate
